# Food Insecurity in Older Adults: Results From the Epidemiology of Chronic Diseases Cohort Study 3

**DOI:** 10.3389/fmed.2018.00203

**Published:** 2018-07-12

**Authors:** Simone G. Fernandes, Ana M. Rodrigues, Carla Nunes, Osvaldo Santos, Maria J. Gregório, Rute Dinis de Sousa, Sara Dias, Helena Canhão

**Affiliations:** ^1^Escola Nacional de Saúde Pública, Universidade Nova de Lisboa, Lisbon, Portugal; ^2^CEDOC, EpiDoc Unit – Unidade de Epidemiologia em Doenças Crónicas, NOVA Medical School, Universidade Nova de Lisboa, Lisbon, Portugal; ^3^Centro de Investigação em Saúde Pública, Escola Nacional de Saúde Pública, Universidade Nova de Lisboa, Lisbon, Portugal; ^4^Instituto de Medicina Preventiva e Saúde Pública, Faculdade de Medicina, Universidade de Lisboa, Lisbon, Portugal; ^5^Instituto de Saúde Ambiental, Faculdade de Medicina, Universidade de Lisboa, Lisbon, Portugal

**Keywords:** food insecurity, chronic diseases, quality of life, older adults, management of chronic diseases

## Abstract

**Introduction:** The public health problem of food insecurity also affects the elderly population. This study aimed to estimate the prevalence of household food insecurity and its associations with chronic disease and health-related quality of life characteristics in individuals ≥65 years of age living in the community in Portugal.

**Methods:** The data were collected from the Epidemiology of Chronic Diseases Cohort Study 3 (EpiDoC3)—Promoting Food Security Study (2015–2016), which was the third evaluation wave of the EpiDoC and represented the Portuguese adult population. Food insecurity was assessed using a psychometric scale adapted from the Brazilian Food Insecurity Scale. The data on sociodemographic variables, chronic disease, and management of chronic disease were self-reported. Health-related quality of life were assessed using the European Quality of Life Survey (version validated for the Portuguese population). Logistic regression models were used to determine crude and adjusted odds ratios (for age group, gender, region, and education). The dependent variable was the perceived level of food security.

**Results:** Among older adults, 23% were living in a food-insecure household. The odds of living in a food-insecure household were higher for individuals in the 70–74 years age group (odds ratio (OR) = 1.405, 95% confidence interval (CI) 1.392–1.417), females (OR = 1.545, 95% CI 1.534–1.556), those with less education (OR = 3.355, 95% CI 3.306–3.404), low income (OR = 4,150, 95% CI 4.091–4.210), and those reporting it was very difficult to live with the current income (OR = 16.665, 95% CI 16.482–16.851). The odds of having a chronic disease were also greater among individuals living in food-insecure households: diabetes mellitus (OR = 1.832, 95% CI 1.818–1.846), pulmonary diseases (OR = 1.628, 95% CI 1.606–1.651), cardiac disease (OR = 1.329, 95% CI 1.319–1.340), obesity (OR = 1.493, 95% CI 1.477–1.508), those who reduced their frequency of medical visits (OR = 4.381, 95% CI 4.334–4.428), and who stopped taking medication due to economic difficulties (OR = 5.477, 95% CI 5.422–5.532). Older adults in food-insecure households had lower health-related quality of life (OR = 0.212, 95% CI 0.210–0.214).

**Conclusions:** Our findings indicated that food insecurity was significantly associated with economic factors, higher values for prevalence of chronic diseases, poor management of chronic diseases, and decreased health-related quality of life in older adults living in the community.

## Introduction

Populations worldwide are undergoing unprecedented changes in age demographics. In Portugal and other European countries, more than 19% of the individuals are ≥65 years of age (1, 2). Age is the most powerful predictor of morbidity and mortality. Multifaceted and numerous mechanisms link age to health status (1). To maximize health and well-being, healthcare systems should be responsive to the diversity and heterogeneity of the health status of older adults (1).

The increasing proportions of countries' oldest populations are associated with greater vulnerability and high-risk of development of chronic diseases and disabilities. These negative health outcomes have direct effects on access to adequate food and result in food insecurity (3). Food security is the condition where “*all people, at all times, have physical and economic access to sufficient, safe and nutritious food to meet their dietary needs and food preferences for an active and healthy life”* (4). This broad concept encompasses the availability of food and the accessibility and proper use of food. Food insecurity can affect the health and well-being of individuals (4–8). Nutrition is one of the main determinants of healthy and active aging. Consumption of nutritious food is crucial for physiological well-being and better health and quality of life (9).

The health characteristics of older adults differ from other age groups. The multidimensional phenomenon of food insecurity is also different in this population (10, 11). In older populations, food insecurity results from more than financial resource constraints. Functional impairment, not owning a home, isolation, gender, financial vulnerability, and poor health have statistically significant associations with food insecurity. These associations suggest that differences in food use between older and younger populations should be considered. These important risk factors for food insecurity tend to occur together, which results in a much higher risk for food insecurity in older populations (8, 10–13). As older populations increase in size, accurate assessments of the extension of food insecurity become more essential for public health (14). Previous studies of food insecurity have mostly examined populations of children and non-elderly adults. Little is known about the characteristics of food insecurity in older adults and associations with health outcomes (11, 15).

Approximately 23% of the global burden of disease is attributable to conditions that affect older people; the main conditions that contribute to this excessive burden are chronic diseases (16). Chronic disease can negatively affect the ability to shop for food, carry foodstuffs home, and prepare meals, which can affect older adults' food security as much as financial vulnerability (5). A poor diet also has severe effects on quality of life and health. Older adults who adopt poor dietary patterns have higher risks of malnutrition, frailty, deteriorating health conditions, and disability compared with those that consume a healthy diet (9, 12). Previous findings indicate that food insecurity is particularly prevalent among older adults (11, 12, 17) and is associated with poor health (5, 12), increased depression, disability (18), and poor quality of life (5, 19).

National and international initiatives promote active and healthy aging, but there are still opportunities for the results of this approach to be reflected in increased quality of life and health of older adults. Rethinking aging implies a true transverse commitment and reconsideration of the entire set of associated factors (20). One of the main objectives of the Healthy People 2020 Report is to “*improve the health, function, and quality of life”* (21), so understanding the associations between food insecurity, chronic diseases, and quality of life is fundamental for improvement of health policies and resulting successful promotion of active and healthy aging populations. The purpose of this study was to estimate the prevalence of household food insecurity and its associations with chronic diseases and health-related quality of life (HRQoL) factors in individuals ≥65 years of age living in the community.

## Materials and methods

This observational study used a cross-sectional analysis of data from a national ongoing prospective cohort study, the Epidemiology of Chronic Diseases Cohort Study (EpiDoC). The data from this population-based study represent the Portuguese adult non-institutionalized (i.e., live in private homes) population in Portugal (Mainland, Madeira, and Azores Islands). The EpiDoC cohort began with the EpiDoC 1 study (EpiReumaPt); two subsequent evaluations have been performed using data from the same subjects [EpiDoC 2 (CoReumaPt) and EpiDoC 3 (Promoting Food Security)] studies. The sampling method used for the EpiDoc 1 study followed a cross-country random route procedure; sampling was stratified by region (North, Centre, Lisbon, Alentejo, Algarve, Madeira, and Azores) and size of locality. Data collection was performed using a computer-assisted telephone interview method. The number of interviews conducted for each stratum was proportional to the actual distribution of the population. Census 2001 results indicated that the eligible Portuguese population ≥18 years old was *N* = 7,719,986. Therefore, a sample of 10,661 participants was randomly selected (random route method) (22). All 10,661 participants in the EpiDoC 1 study signed an informed consent form for follow-up, and those who provided their telephone number were included in the subsequent EpiDoC cohort follow-up evaluations (EpiDoC 2 and EpiDoC 3). In the EpiDoC 2 and EpiDoC 3 studies, all participants were contacted by telephone (23, 24). Of the 9,003 participants eligible for the EpiDoC 3 (Promoting Food Security) study, 2,366 were lost due to unsuccessful contact and 1,004 participants were lost to follow-up for other reasons; there were 5,653 participants in the third wave of the study. A total of 1,885 individuals in this Portuguese noninstitutionalised population were ≥65 years of age; the data from these participants were included in the analyses described in this article.

### Instruments and variables

Sociodemographic (age, gender, years of education, and health region) and socioeconomic (income and information on perception of family income) data were collected. Data on self-reported diseases (high cholesterol levels, hypertension, diabetes, gastrointestinal disease, mental disease, cardiac disease, pulmonary disease, cancer, neurological disease, hyperuricemia, and urinary disease) were also assessed. HRQoL was assessed using the European Quality of Life Survey (version validated for the Portuguese population) (25) with five dimensions and three levels (EQ-5D-3L). The EQ-5D descriptive system was converted into a single index using a formula that assigned values to each of the levels in each dimension. These value-sets were derived for use of the EQ-5D for the Portuguese population (25). A higher EQ-5D index score corresponded to a higher quality of life. Self-perception of general health status at the time of the survey was also assessed by adapting the original EQ5D-3L visual analog scale to the question “*Considering a scale on which the best state of health you can imagine is 100 and the worst state of health you can imagine is 0, we would like you to tell us how good or bad it is, in your opinion, your state of health today?”*.

Data on whether there was a reduction in the numbers of visits to a physician due to economic difficulties (Yes/No), whether the respondent took any medication (Yes/No), and whether the respondent stopped taking any medication due to economic difficulties (Yes/No) were also recorded. Body mass index (BMI, body weight in kilograms divided by the square of the height in meters) was calculated using the values for self-reported weight and height. The BMI values were assigned to categories according to World Health Organization criteria (26).

Food insecurity was assessed using a psychometric scale adapted from the Brazilian Food Insecurity Scale; this scale was adapted from the US Household Food Security Survey Module (27–29). This tool was used to assess the quantitative and qualitative components of food insecurity within the 3 months before the respondent answered the survey. A score ranging from 0 to 14 was obtained as an outcome of the total number of affirmative answers. Each score was used to assign the respondent to one of four categories of food insecurity (i.e., “food security,” “low food insecurity,” “moderate food insecurity,” and “severe food insecurity”; Table 1).

**Table 1:** Definitions of Food Security levels.

**Food Security Level**	**Cut-offs**	**Definition**
Food Security	If the household includes minors (<18 years old): 0 Household without minors (≥18 years): 0	Households show access at all times to enough food for an active and healthy life.
Low Food Insecurity	If the household includes minors (<18 years old): 1 - 5 Household without minors (≥18 years): 1 – 3	Households reports at least anxiety about lack of food to meet dietary needs. At this level, coping strategies to deal with economic and food constraints can also have an important on the reduction of diet quality.
Moderate Food Insecurity	If the household includes minors (<18 years old): 6 - 9 Household without minors (≥18 years): 4 – 5	Adults in the household reported food intake reduction and changes in eating patterns due to economic difficulties in accessing food.
Severe Food Insecurity	If the household includes minors (<18 years old): 10 - 14 Household without minors (≥18 years): 6 – 8	At this level, households without children experienced the physical sensation of hunger and households with children reported a reduction of children's food intake.

*Adapted from the Brazilian Institute of Geography and Statistics. National Household Sample Survey - Food Security 2004/2009. Rio de Janeiro; 2010 (30)*.

### Statistical analysis

The statistical analysis was performed using the Statistical Package for the Social Sciences - IBM-SPSS for Windows 24.0®. To assure the representativeness of the sample for the 65 years and older Portuguese population, weighting coefficients were computed and used for the additional statistical analyses. The EpiDoC 3 study participants and non-participants were first compared regarding sociodemographic, socioeconomic, and health status characteristics. We then adjusted the weights based on the corresponding population stratification groups (gender, age group, and health region). Weighted proportions of food insecurity according to age group, gender, health region, years of education, income, and household income perception were calculated. Prevalence estimates for food insecurity were calculated as weighted proportions, consistent with the sample design. After the descriptive analyses were performed, each participant was assigned to the “Food Security” or “Food Insecurity” (including mild, moderate and severe food insecurity) categories to evaluate the relationships between food insecurity and the other study variables. The magnitudes of the associations between the variables were calculated using binary logistic regression models. The dependent variable was food security status [“food insecurity” (event) vs. “food security”]. Independent variables were sequentially added to the model based on existing knowledge about the variables that could affect the event. Crude and adjusted odds ratios (ORs; age group, gender, health region, and years of education) and the corresponding 95% confidence intervals (CIs) were computed. EQ5D-3L and self-perception of general health status data were analyzed as continuous variables. We interpreted these variables considering that the EQ5D-3L values ranged from 0 to 1 and that the self-perception of general health status values ranged from 0 to 100. A significance level of α = 0.05 (two-tailed) was used for all analyses.

### Ethical issues

The EpiDoC 3 study was performed according to the criteria established by the Declaration of Helsinki and revised in 2013 in Fortaleza (31). The study was reviewed and approved by the National (Portuguese) Committee for Data Protection and by the NOVA Medical School Ethics Committee. The participants gave informed signed consent to be included in all phases of the study.

## Results

The data were collected using telephone interviews of 1,885 Portuguese people ≥ 65 years of age living in communities in the mainland, or the Madeira or Azores islands, between July 2015 and September 2016. The sample consisted of 55.5% women and 44.5% men (mean ± standard deviation (SD), 74.3 ± 6.8 years (range, 65–104 years). The results for the sociodemographic and socioeconomic characteristics of this older population are presented in Table 2.

**Table 2:** Sociodemographic and socioeconomic characteristics of older adults.

**Variables**	**Total *n* (%)**
**AGE (*****n*** = **1885)**	
65-69 years	563 (29.4)
70-74 years	475 (27)
75-79 years	393 (19.9)
≥80 years	454 (23.7)
**GENDER (*****n*** = **1885)**	
Female	1243 (55.5)
Male	642 (44.5)
**EDUCATION LEVEL (*****n*** = **1877)**	
0 years of education	174 (9.7)
1 - 4 years of education	1205 (63.8)
5 – 9 years of education	267 (13.9)
10- 12 years of education	117 (6.3)
>12 years of education	114 (6.4)
**HEALTH REGION (*****n*** = **1885)**	
North	501 (32.2)
Centre	414 (27.1)
Lisbon	371 (23.1)
Alentejo	136 (9.6)
Algarve	78 (4.1)
Azores	183 (1.8)
Madeira	202 (2)
**INCOME (*****n*** = **1384)**	
≤ 500€	950 (64.4)
501€ until 750€	208 (15.5)
751€ until 1000€	89 (9.6)
1001€ until 1500€	84 (6.4)
≥1501€	53 (4.1)
**INCOME PERCEPTION (*****n*** = **1869)**	
I live comfortably with my current income	223 (12.4)
I can live with my current income	777 (41.9)
It is difficult to live with the current income	585 (30.7)
It is very difficult to live with the current income	284 (14.9)

*All percentages were weighted for correcting to population representativeness*.

### Prevalence of food insecurity

A Cronbach's α coefficient of 0.949 was found when we analyzed only the adult household-related items of the scale. The first validation of the Household Food Insecurity Scale used a sample from the Portuguese population. It was performed by Gregorio et al. in 2015. They found a Cronbach's α coefficient of 0.865 (27).

Twenty-three percent (*n* = 500) of 65 years and older households reported being food-insecure. Of this food-insecure group, 16.3% were in the low food insecurity, 4.8% were in the moderate, and 2% were in the severe food insecurity, group (Table 3). There were apparent differences between genders and age groups (Table 4). Older females were more likely to report living in a food-insecure household (26.5%) compared with older males (18.9%) (OR = 1.545, 95% CI 1.534–1.556). A higher proportion (25.9%) of food insecurity was found in the households of the individuals in the 70–74 years age group (OR = 1.405, 95% CI 1.392–1.417). The highest proportion of severe food insecurity was also found among the individuals in the 70–74 years age group (2.9%) (Table 3).

**Table 3:** Food Insecurity status by age group and gender.

**Variables**	**Total *n* (%)**	**Food Security *n* (%)**	**Low Food Insecurity *n* (%)**	**Moderate Food Insecurity *n* (%)**	**Severe Food Insecurity *n* (%)**
**AGE (*****n*** = **1885)**					
65-69 years	563 (29.4)	418 (80.1)	94 (14.1)	33 (4.0)	18 (1.8)
70-74 years	475 (27)	347 (74.1)	85 (19.4)	26 (2.6)	17 (2.9)
75-79	393 (19.9)	288 (76.4)	68 (16.6)	32 (6.1)	5 (0.9)
≥80 years	454 (23.7)	332 (76.5)	80 (15.1)	30 (6.1)	12 (2.2)
**GENDER (*****n*** = **1885)**					
Female	1243 (55.5)	865 (73.5)	241 (17.1)	93 (6.2)	44 (3.1)
Male	642 (44.5)	520 (81.1)	86 (15.1)	28 (3.1)	8 (0.7)

*All percentages were weighted for correcting to population representativeness*.

**Table 4:** Food Insecurity status: Associations with Sociodemographic Characteristics.

**Variables**	**Total *n* (%)**	**Food Security**	**Food Insecurity**	**Crude OR (95% CI)**
		***n* (%)**	***n* (%)**	
**AGE (*****n*** = **1885)**				
65-69 years	563 (29.4)	418 (80.1)	155 (19.9)	1
70-74 years	475 (27)	347 (74.1)	128 (25.9)	1.405(1.392-1.417)
75-79 years	393 (19.9)	288 (76.4)	105 (23.6)	1.246 (1.233-1.258)
≥80 years	454 (23.7)	332 (76.5)	122 (23.5)	1.234 (1.222-1.246)
**GENDER (*****n*** = **1885)**				
Female	1243 (55.5)	865 (73.5)	378 (26.5)	1.545 (1.534-1.556)
Male	642 (44.5)	520 (81.1)	122 (18.9)	1
**EDUCATION LEVEL (*****n*** = **1877)**				
0 years of education	174 (9.7)	91 (65.6)	83 (34.4)	3.355 (3.306-3.404)
1 - 4 years of education	1205 (63.8)	852 (73.8)	436 (26.2)	2.269 (2.242-2.296)
5 – 9 years of education	267 (13.9)	223 (86.5)	44 (13.5)	1
10- 12 years of education	117 (6.3)	105 (89.2)	12 (10.2)	0.776 (0.760-0.793)
>12 years of education	114 (6.4)	108 (91.8)	6 (8.2)	0.572 (0.559-0.585)
**HEALTH REGION (*****n*** = **1885)**				
North	501 (32.2)	359 (75.6)	142 (24.4)	1
Centre	414 (27.1)	312 (76.1)	102 (23.9)	0.972 (0.964-0.980)
Lisboa	371 (23.1)	312 (81.2)	59 (18.8)	0.717 (0.710-0.724)
Alentejo	136 (9.6)	104 (78.4)	32 (21.6)	0.853 (0.842-0.864)
Algarve	78 (4.1)	57(77.8)	21 (22.2)	0.881 (0.866-0.897)
Azores	183 (1.8)	123 (68.1)	60 (31.9)	1.451 (1.417-1.485)
Madeira	202 (2)	118 (58.8)	84 (41.2)	2.161 (2.116-2.207)

*All percentages were weighted for correcting to population representativeness*.

## Variables associated with food insecurity

### Sociodemographic characteristics

The results for the analysis of the sociodemographic characteristics of the sample and for the application of a binary logistic regression model for the event (food insecurity, binary variable) are presented in Table 4. The results indicated that for the sociodemographic factors related to food insecurity, females (OR = 1.545, 95% CI 1.534–1.556), the oldest ages (70–74 years vs. 65–69 years: OR = 1.405, 95% CI 1.392–1.417; 75–79 years vs. 65–69 years: OR = 1.246, 95% CI 1.233–1.258; ≥80 years vs. 65–69 years: OR = 1.234, 95% CI 1.222–1.246), lower levels of education (0 years of school vs. 5–9 years: OR = 3.355, 95% CI 3.306–3.404; 1–4 years vs. 5–9 years: OR = 2.269, 95% CI 2.242–2.296), living in the Azores (Azores vs. North: OR = 1.451, 95% CI 1.417–1.485) and Madeira (Madeira vs. North: OR = 2.161, 95% CI 2.116–2.207) were associated with increased odds of living in a food-insecure household.

### Socioeconomic characteristics

The results for the application of a binary logistic regression model for the event food insecurity are presented in Table 5. The socioeconomic characteristics were included in the model and the results were presented as crude and adjusted ORs (i.e., adjusted by age group, gender, education level, and health region). The analysis of socioeconomic characteristics revealed that having an monthly income ≤ 500€ (i.e., ≤ 500€ vs. 501€ to 750€: OR = 3.125, 95% CI 4.091–4.210) and reporting having financial difficulties (*It is difficult to live with the current income* vs. *I can live with my current income*: OR = 6.366, 95% CI 6.304–6.429) or reporting that it was very difficult to live with the current income (*It is very difficult to live with the current income* vs. *I can live with my current income*: OR = 16.665, 95% CI 16.482–16.851) were associated with increased odds of living in a food-insecure household.

**Table 5:** Food Insecurity status: Associations with Socioeconomic Characteristics.

**Variables**	**Total *n* (%)**	**Food Security**	**Food Insecurity**	**Crude OR (95% CI)**	Adjusted OR^*^ (95% CI)
		***n* (%)**	***n* (%)**		
**INCOME (*****n*** = **1384)**					
≤ 500€	950 (64.4)	614 (67.2)	336 (32.8)	4.150 (4.091-4.210)	4.442 (4.377-4.508)
501€ until 750€	208 (15.5)	177 (89.5)	31 (10.5)	1	1
751€ until 1000€	89 (9.6)	81 (94.9)	8 (5.1)	0.457 (0.444-0.470)	0.510 (0.496-0.525)
1001€ until 1500€	84 (6.4)	78 (92.4)	6 (7.6)	0.701 (0.681-0.721)	0.978 (0.950-1.008)
≥1501€	53 (4.1)	51 (89.5)	2 (10.5)	1.000 (0.971-1.031)	1.267 (1.226-1.310)
**INCOME PERCEPTION (*****n*** = **1869)**					
I live comfortably with my current income	223 (12.4)	217 (94.6)	6 (5.4)	0.688 (0.675-0.702)	0.774 (0.759-0.790)
I can live with my current income	777 (41.9)	703 (92.4)	74 (7.6)	1	1
It is difficult to live with the current income	585 (30.7)	359 (65.5)	226 (34.5)	6.366 (6.304-6.429)	6.228 (6.166-6.291)
It is very difficult to live with the current income	284 (14.9)	92 (42.0)	192 (58.0)	16.665 (16.482-16.851)	15.817 (15.637-15.998)

* Adjusted Odds Ratio to gender, age, education level, and health region.

*All percentages were weighted for correcting to population representativeness*.

### Chronic diseases

The results for the crude and adjusted ORs (i.e., adjusted for age group, gender, education level, and health region) for the presence of household food insecurity and chronic disease are presented in Table 6. The results indicated that older adults living in food-insecure households had greater odds of having one or more chronic diseases (OR = 1.161, 95% CI 1.158–1.164). The chronic conditions were diabetes (OR = 1.832, 95% CI 1.818–1.846), hypercholesterolemia (OR = 1.138, 95% CI 1.129–1.146), pulmonary disease (OR = 1.628, 95% CI 1.606–1.651), cardiac disease (OR = 1.329, 95% CI 1.319–1.340), digestive disease (OR = 1.259, 95% CI 1.247–1.271), mental disease (OR = 1.334, 95% CI 1.322–1.347), and urinary disease (OR = 1.566, 95% CI 1.550–1.582). Except for the variable hypercholesterolemia (ORa = 0.992, 95% CI 0.988–1.004), these associations were present before and after adjusting the model. For the variable BMI, older adults from food-insecure households had greater odds of pre-obesity (pre-obesity vs. normal weight: OR = 1.364, 95% CI 1.353–1.376) and obesity (obesity vs. normal weight: OR = 1.493, 95% CI 1.477–1.508), and lower odds of being underweight (underweight vs. normal weight: OR = 0.572, 95% CI 0.543–0.603), before and after adjusting for age group, gender, education level, and health region.

**Table 6:** Food Insecurity status: Associations with Chronic Diseases.

**Variables**	**Food Security *n* (%)**	**Food Insecurity *n* (%)**	**Crude OR (95% CI)**	Adjusted OR^*^ (95% CI)
**CHRONIC DISEASES**				
**Number of Chronic Diseases**	Mean±SD: 1.98 ± 1.41	Mean±SD: 2.31 ± 1.66	1.161 (1.158-1.164)	1.128 (1.125-.130)
**DIABETES MELLITUS (*****n*** = **1626)**				
No	910 (79.9)	294 (20.1)	1	1
Yes	286 (68.4)	136 (31.6)	1.832 (1.818-1.846)	1.764 (1.750-1.778)
**HYPERCHOLESTEROLEMIA (*****n*** = **1568)**				
No	518 (77.8)	162 (22.2)	1	1
Yes	631 (75.5)	257 (24.5)	1.138 (1.129-1.146)	1.992 (0.988-1.004)
**PULMONARY DISEASES (*****n*** = **1644)**				
No	1130 (77.3)	405 (22.7)	1	1
Yes	76 (67.6)	33 (32.4)	1.628 (1.606-1.651)	1.611 (1.588-1.634)
**CARDIAC DISEASES (*****n*** = **1619)**				
No	887 (78.4)	286 (21.6)	1	1
Yes	306 (73.2)	140 (26.8)	1.329 (1.319-1.340)	1.298 (1.287-1.308)
**DIGESTIVE DISEASES (*****n*** = **1640)**				
No	1029 (77.6)	358 (22.4)	1	1
Yes	178 (73.4)	75 (26.6)	1.259 (1.247-1.271)	1.296 (1.283-1.309)
**MENTAL DISEASES (*****n*** = **1653)**				
No	967 (77.8)	343 (22.2)	1	1
Yes	246 (72.4)	97 (27.6)	1.334 (1.322-1.347)	1.245 (1.233-1.257)
**ONCOLOGIC DISEASES (*****n*** = **1654)**				
No	1089 (76.8)	393 (23.2)	1	1
Yes	124 (77.0)	48 (23.0)	0.988 (0.977-1.000)	1.040 (1.028-1.052)
**URINARY DISEASES (*****n*** = **1621)**				
No	1065 (77.9)	396 (22.1)	1	1
Yes	126 (69.2)	34 (30.8)	1.566 (1.550-1.582)	1.994 (1.972-2.016)
**BODY MASS INDEX CLASSIFICATION**				
BMI CLASSES^**^ **(*****n*** = **1634)**				
Underweight	15 (88.7)	2 (11.3)	0.572 (0,543-0,603)	0.421 (0.400-0.443)
Normal weight	375 (81.7)	110 (18.3)	1	1
Pre-obesity	579 (76.6)	180 (23.4)	1.364 (1.353-1.376)	1.388 (1.376-1.401)
Obesity	271 (75.0)	102 (25.0)	1.493 (1.477-1.508)	1.314 (1.300-1.328)

* *Adjusted Odds Ratio to gender, age, education level, and health region*.

*All percentages and means were weighted for correcting to population representativeness*.

** BMI Classes: Underweight < 18,5kg/m^2^; Normal weight 18,5-24,9kg/m^2^; Pré-obesity 25-29,9kg/m^2^; Obesity ≥30kg/m^2^

### Management of chronic diseases

The results for the application of a binary logistic regression model for the event food insecurity are presented in Table 7. The variables related to management of chronic disease were included in the model, and the results were presented as crude and adjusted ORs (i.e., adjusted for age group, gender, education level, and health region). The results of the analysis of the difficulties experienced by older adults in the management of these chronic diseases indicated that those who reported a decrease in medical visits or who stopped taking medication due to economic reasons had a higher odds (OR = 4.381, 95% CI 4.334–4.428, and OR = 5.477, 95% CI 5.422–5.532, respectively) of living in a food-insecure household, before and after adjustment of the model.

**Table 7:** Food Insecurity status: Associations with Management of Chronic Diseases.

**Variables**	**Food Security *n* (%)**	**Food Insecurity *n* (%)**	**Crude OR (95% CI)**	Adjusted OR^*^ (95% CI)
**REDUCTION OF MEDICAL VISITS DUE TO ECONOMIC DIFFICULTIES (*****n*** = **1883)**				
No	1305 (79.4)	393 (20.6)	1	1
Yes	78 (46.9)	107 (53.1)	4.381 (4.334-4.428)	4.350 (4.301-4.398)
**TAKE MEDICATION (*****n*** = **1882)**				
No	103 (76.2)	36 (23.8)	-	-
Yes	1280 (77.0)	463 (23.0)		
**STOP TAKING MEDICATION DUE TO ECONOMIC DIFFICULTIES (*****n*** = **1875)**				
No	1297 (80.4)	372 (19.6)	1	1
Yes	80 (42.8)	126 (57.2)	5.477 (5.422-5.532)	5.152 (5.099-5.207)

* Adjusted Odds Ratio to gender, age, health region, and level of education.

*All percentages were weighted for correcting to population representativeness*.

### Health related quality of life

The results for the crude ORs and adjusted ORs (i.e., adjusted for age group, gender, education level, and health region) for the presence of household food insecurity and HRQoL are presented in Table 8. The results indicated that before and after adjustment of the model, older adults from food-insecure households had higher odds of a lower HRQoL (EQ5D-3L score: OR = 0.212, 95% CI 0.210–0.214). The analysis of the self-perception of general health status (thermometer scale from the EQ5D-3L questionnaire adapted to one question) revealed that older adults who reported a better health status had lower odds of living in a food-insecure household, before and after adjusting the model (OR = 0.976, 95% CI 0.976–0.976).

**Table 8:** Food Insecurity status: Associations with Health-related Quality of Life.

**Variables**	**Food Security**	**Food Insecurity**	**Crude OR (95% CI)**	Adjusted OR^*^ (95% CI)
Health-Related Quality of Life - EQ5D-3L score (*n* = 1715)	Mean ± SD: 0.683 ± 0.318 Medium: 0.693 Minimum: −0.438 Maximum: 1	Mean ± SD: 0.511 ± 0.336 Medium: 0.536 Minimum: −0.344 Maximum: 1	0.212 (0.210–0.214)	0.251 (0.248–0.254)
Self-perception of general health status (*n =* 1715)	Mean ± SD: 70.06 ± 19.79 Medium: 70 Minimum: 0 Maximum: 100	Mean ± SD: 59.33 ± 22.02 Medium: 60 Minimum: 0 Maximum: 100	0.976 (0.976–0.976)	0.979 (0.978–0.979)

* Adjusted Odds Ratio to gender, age, education level, and health region.

*All means were weighted for correcting to population representativeness*.

## Discussion

This study aimed to estimate the prevalence of food insecurity and its association with chronic diseases and HRQoL in individuals ≥65 years of age living in households in the community. To our knowledge, a study with these objectives has not been published.

We found a food insecurity prevalence of 23%. Few studies of food insecurity in Portugal have been performed, and none have specifically examined the older adult population. A national study of the EpiDoc cohort revealed a food insecurity prevalence of 19.3% in the adult population (≥18 years) (23). The results of this current study indicated that this problem is more prevalent in the older population compared with the adult population. Other analyses adult populations found values for prevalence of food insecurity between 8.1% and 50.7% (32–34). However, comparing the results of our study and published studies should be done carefully because the methodological approaches, sources of collected data, and the times of data collection were different.

Similarly, direct comparison of the values for prevalence found in our study with estimates from other countries is not always feasible because the methods of data collection differ. However, some studies used similar methodological approaches to our study. The results of a study performed by Goldberg et al. of a sample of 2,045 older adults ≥60 years of age who were included in the National Health and Nutrition Examination Survey (NHANES, USA) 2007–2008 indicated that >9% of older adult households were food-insecure (35). Russell et al. found that of a sample of 3,509 older adults in Australia, 13% reported food insecurity (5). Other studies performed in United States and Australian populations revealed a higher prevalence of food insecurity in disadvantaged urban areas (6, 8, 36). Compared with our study, these two studies found lower values for prevalence of food insecurity. However, the differences might be due to differences in the participants' ages (i.e., the other studies included participants <65 years of age). The differences could also be due to differences in the sources of collected data (i.e., national vs. regional samples), the socioeconomic disparities between each location (37–39), the socio-economic characteristics of the population (40) or because our sample was collected after a period of economic crisis (41). The data used for other studies were collected before the period of global economic recession (42).

Consistent with the results of previous studies, our study revealed that older adults in food-insecure households were more likely to be women, have less education, and live in households with a lower per capita income (5, 11, 12, 34, 37, 43–45). The income perception is the factor with the most robust association with food insecurity in older adults; it clearly represents the relationship between economic factors and food insecurity (5, 13, 46).

Our findings for the self-reported noncommunicable diseases suggested that older adults with chronic diseases (i.e., diabetes, pulmonary disease, cardiac disease, digestive disease, mental disease, and urinary disease) had increased odds of living in a food-insecure household. Other studies have also found associations between food insecurity and higher values for prevalence of diabetes (34, 47–51), pulmonary disease (51), mental disease (12, 18, 48, 52, 53), cardiac disease (48, 54, 55) and poor health outcomes (56). The higher prevalence can be explained by the results of a study that found that food insecurity requires changes in the household food supply that reduce diet quality (57). Disturbance of the nutrient and dietary patterns linked with food insecurity may clarify the higher rates of chronic illness experienced by food-insecure individuals (55, 57).

There are other putative reasons for the higher prevalence of chronic diseases in older adults from food-insecure households. For example, a review performed by Gucciardi et al. revealed that due to the cost of out-of-pocket health care expenditures such as purchase of prescribed medications and supplies, food insecurity affects compliance with the self-management recommendations given to individuals with diabetes (50). Seligman et al. found that persons in food-insecure households may replace the consumption of healthy foods with less expensive foods that have poor nutritional value and a higher energy density. These individuals will then consume in excess of their energy needs and these intakes can be associated with the development of diabetes (49). This finding is consistent with the finding of another study of older adults (58).

The significant association between food insecurity and pulmonary disease revealed by our study is consistent with the results of studies performed in other countries that indicate that being a smoker is a predictor for reporting characteristics associated with food insecurity (5, 51).

Our study also revealed that a higher proportion of older adults from food-insecure households reported lower rates of being underweight and higher rates of pre-obesity and obesity (Table 6). The results of previous studies have been inconsistent in this area, especially in the older adult populations where this association is weak (12, 45, 55, 59–61). Consistent with our findings, some studies found that food insecurity is associated with greater BMI values (pre-obesity and obesity) in older adults (18, 62). However, other studies have not found this association (12) or it has only been found in populations of older females but not in populations of older males (45, 61). These conflicting results may be related to the evidence that compared with direct assessment, self-reported measures of weight and height in older adults could give biased results (18, 45, 63). The aging process is accompanied by modifications in body composition (e.g., reductions in height resulting from compression of the vertebrae, loss of muscle tone, and variations in body weight). Older adults tend to report the values they maintained in adulthood (18, 45, 60, 64). Therefore, these findings suggest that the food-insecurity association with obesity reported among adults may have different characteristics in older adults.

Some studies have found that food-insecure older adults report lower investment in their health (8). Food insecurity, cost-related medication nonadherence, and a decrease in medical visits are three related problems with negative consequences and public health implications (65). Our study found that older adults who reported a decrease in medical visits or who stopped taking medication for economic reasons had 4.5 and 5.5 higher odds, respectively, of living in a food-insecure household.

Our findings suggested that those who reported that they stopped taking medication due to economic reasons had higher odds of living in a food-insecure household. In Portugal, the acquisition of medication is associated with different levels of reimbursement by the state (range, 15% to 95%). There is typically some expenditure by the elderly for medication. Elderly people who must purchase several medications may not be able to do so, especially if they have a low income. Afulani et al. assessed the association between food security and cost-related medication under use in a sample of 10,401 American older adults (National Health Interview Survey 2011–2012). Consistent with our findings, they found that older adults from food-insecure households were more likely to report cost-related medication under use than the adults living in food-secure households (66). Bengle et al. examined the relationship between cost-related medication nonadherence and food insecurity in a sample of 1,000 low-income Georgian older adult participants of the Older Americans Act Nutrition Program. They found that food-insecure participants were three times more likely to practice cost-related medication nonadherence than their counterparts (65). In a 2008 study of older Georgians, Bhargava et al. found that food-insecure individuals were more likely to report a poorer health status, had more chronic disease, and tended to have lower health care expenditures compared with their counterparts with similar health status. Taken together, these findings suggest that food-insecure older adults may be unable to meet healthcare needs and practice healthy food consumption behaviors (6). Other studies have found similar relationships between food insecurity and lack of adherence to medical treatments and drug therapies (8, 13, 34, 50).

In older adults, food insecurity and higher prevalence of chronic diseases contribute to declines in functionality and corresponding decreases in quality of life (9, 19). The relationships between nutrition and morbidity and mortality in aging are well-established, but more research on HRQoL and food insecurity in older adults is needed. Our findings indicated that older adults from food-insecure households reported a poor HRQoL (Table 8). These findings are consistent with those of a study of an older Australian cohort of community-living persons ≥49 years of age; this study revealed associations between food insecurity and poor HRQoL characteristics (19). Older adults living in food-insecure households report at least some inability to obtain enough food due to economic limitations and that consequently they reduced their diet quality or variety (28, 67). Diet quality and variety are significant determinants of HRQoL in the aging population (68, 69).

Our study also found that older adults from food-insecure households self-reported a lower health status value (Table 8). This finding is consistent with studies that found that than compared with food-secure older adults, food-insecure older adults self-report a poor health status (5, 8, 13, 65).

Our study had some limitations. First, it was based on a cross-sectional analysis that limited the ability to explore casual relationships and establish the temporal sequence of associations. Second, the method used to control the potential effects of sociodemographic variables was based on self-reported conditions, which might contributed to underestimates of the diagnosis of chronic conditions and BMI values (12, 18, 45, 63). Third, the food insecurity scale “*evaluates the food security situation of the household members as a set and not necessarily the condition of any specific household member”* (28, 29). Thus, it was not possible to define the food insecurity status of each older adult living in the household. Despite these limitations, our findings are a valuable resource in understanding key health-related factors associated with food insecurity in older adults. They can be used during development of preventive public health strategies and policies. To our knowledge, this study is one of the first to examine the prevalence of household food insecurity and its associations with chronic diseases and HRQoL of older adults living in the community. The strengths of our study include the use of a randomly selected sample that represented the population of Portugal. The instrument used to record information on food insecurity had high internal consistency measures for this sample. Our study also examined a relationship that has been overlooked in older populations: the association between management of chronic diseases and food insecurity. The results of this study contribute to understanding the inter-related issues that affect health status and disease evolution.

## Conclusions

In conclusion, our results have implications for clinical practice and public policy development. For an increasingly aging population, the greater odds of food insecurity among households of older adults and the associated factors indicates the importance of considering this problem as one of the main public health challenges.

Ensuring that older adults have enough food to meet their needs may be an important way to help them enjoy good health and remain active while aging. Food insecurity in older adults is an undesirable problem that requires additional attention. Food insecurity is associated with higher odds of chronic disease, poor self-management of chronic disease, and lower HRQoL. Because it is determined by economic factors, food insecurity is socially and ethically unacceptable. Therefore, during a period marked by significant financial stress, implementation of strategies aimed at ensuring the food security of the aging population is needed. We suggest that other evaluation and monitoring studies should be performed and further investigation using longitudinal data are needed to determine the health consequences of food insecurity among older adults. These results would assist health professionals and policymakers to better understand the barriers to achieve improved health in this population.

## Ethics statement

EpiDoC 3 study was performed according to the principles established by the Declaration of Helsinki and revised in 2013 in Fortaleza. The study was reviewed and approved by the National Committee for Data Protection and by the NOVA Medical School Ethics Committee. Participants provided informed consent to contribute in all phases of the study.

## Author contributions

All authors contributed to this work. HC, AR, and MG performed the initial study. SF and CN analyzed the data. SF, CN, and OS interpreted the data. SF wrote the manuscript. HC, AR, and MG provided scientific support and revised the manuscript. All authors contributed to the further discussion of the manuscript. All authors read and approved the final manuscript.

### Conflict of interest statement

The authors declare that the research was conducted in the absence of any commercial or financial relationships that could be construed as a potential conflict of interest.

## Abbreviations

EpiDoC3: Promoting Food Security Study
EpiDoC cohort: Epidemiology of Chronic Diseases Cohort Study
BMI: Body mass index
HRQoL: Health-related quality of life
EQ-5D-3L: European Quality of Life Survey with five dimensions and three levels
EQ VAS: thermometer scale from the EQ5D-3L questionnaire.
